# Physicochemical Aspects of Mixed Micelle Formation Between Amphiphilic Drugs and Surfactants

**DOI:** 10.3390/ijms27125400

**Published:** 2026-06-15

**Authors:** Ádám Juhász, Bianka Torma, Egon F. Várkonyi, László Seres, Norbert Varga, Árpád Turcsányi, Edit Csapó

**Affiliations:** 1 MTA-SZTE Lendület “Momentum” Noble Metal Nanostructures Research Group, University of Szeged, Rerrich B. Sq. 1, H-6720 Szeged, Hungary; tormabianka@chem.u-szeged.hu (B.T.); f.varkonyiegon@chem.u-szeged.hu (E.F.V.); seres.laszlo@chem.u-szeged.hu (L.S.); vargano@chem.u-szeged.hu (N.V.); tarpad@chem.u-szeged.hu (Á.T.); 2 Interdisciplinary Excellence Center, Department of Physical Chemistry and Materials Science, University of Szeged, Rerrich B. Sq. 1, H-6720 Szeged, Hungary

**Keywords:** amphiphilic drugs, surfactants, solubilization, mixed micelles

## Abstract

The rational design of mixed micellar systems has emerged as a cornerstone of modern nanomedicine, offering unprecedented control over the solubility and bioavailability of challenging therapeutic agents. This review provides a comprehensive analysis of the physicochemical principles governing the assembly of amphiphilic drugs and surfactants into synergistic nanostructures. By articulating the transition from traditional guest/host solubilization to “drug-as-component” models, we highlight the critical role of molecular interactions in achieving therapeutic precision. It further outlines the experimental methodologies used to investigate these systems and elucidates how they enhance the solubility, stability, and bioavailability of poorly water-soluble drugs. Special emphasis is placed on the practical applications of synergy in reducing systemic toxicity and optimizing drug release kinetics, providing a roadmap for the development of next-generation nano-pharmaceuticals. The functionality of these systems is significantly influenced by the molecular interactions among their constituents; thus, quantitative analysis of these interactions might enhance the formulation of more effective pharmaceuticals. This review outlines the key physicochemical principles of mixed micelle formation, including thermodynamics and synergistic interactions of amphiphiles, while emphasizing their relevance in current research and practical pharmaceutical applications. Various experimental methods, such as surface tension measurement, conductometric and calorimetric tests, and spectroscopic techniques, are compared in terms of their conditions of application and performance in understanding micelle formation and micelle structure. We clearly point out that the interpretation and evaluation of the properties of colloidal systems containing drug molecules solubilized by mixed micelles and an amphiphilic drug incorporated into micelles must be discussed and evaluated separately. Understanding the limitations and characteristics of the physical/chemical principles applied is essential for the rational design of mixed micelle carriers tailored to specific therapeutic needs.

## 1. Introduction

The evolution of colloid chemistry from simple micelle models to sophisticated drug delivery platforms reflects a broader shift towards precision pharmaceutics. Since the pioneering work of McBain in the early 20th century, our understanding of self-assembly has matured from observing bulk transport phenomena to engineering molecular-level synergy. Today, mixed micelles represent not just solubilizers, but dynamic carriers capable of bypassing biological barriers and enhancing the therapeutic index of diverse drug classes.

Micellar systems play a pivotal role in the field of associated amphiphiles and interface science, serving as model systems for understanding self-assembly, solubilization, and molecular interactions in aqueous media [1]. In pharmaceutical sciences, micelles formed by surfactants have long been employed to enhance the solubility of poorly water-soluble drugs, thereby improving their bioavailability and therapeutic efficacy [2,3]. However, the incorporation of amphiphilic drugs [4] into micellar structures introduces an additional level of complexity and functional diversity. Such amphiphilic drugs can simultaneously act as solubilizers and co-surfactants, modifying micellar properties and aggregation [4,5].

Mixed micelle formation between amphiphilic drugs and surfactants has gained growing interest due to the observed synergistic effects that often result in reduced critical micelle concentration (*cmc*), enhanced solubilization capacity, and modified thermodynamic stability [6]. Understanding these cooperative interactions requires a detailed physicochemical approach encompassing both experimental and theoretical perspectives. Parameters such as mixed micelle composition, activity coefficients, and interaction parameters provide valuable insight into the nature of drug–surfactant associations [7,8]. Synergism in mixed micelles occurs when the combination of distinct surfactants produces aggregates with physicochemical properties (such as lower *cmc* and enhanced thermodynamic stability) that exceed those of their individual components. This non-ideal, cooperative behavior arises from complementary molecular characteristics like headgroup charge and hydrophobic tail length, which facilitate more efficient packing and reduced intermolecular repulsion [1,9]. In pharmaceutical applications, these systems are critical for solubilizing poorly water-soluble drug candidates, as they allow for the rational engineering of micellar cores with optimized polarity and fluidity [10]. By maintaining structural integrity upon physiological dilution, mixed micelles prevent premature drug precipitation and offer precise control over release kinetics. Furthermore, the high solubilization efficiency achieved at lower total surfactant concentrations improves biocompatibility and reduces systemic toxicity. Ultimately, this synergistic approach provides a versatile platform for the delivery of challenging therapeutic agents, offering a level of stability and membrane permeability unattainable by single-surfactant systems [11,12].

The aim of this review is to summarize the fundamental physicochemical aspects governing the formation and stability of mixed micelles composed of amphiphilic drugs and surfactants. It is important to note that the present review focuses primarily on the equilibrium aspects of mixed micellization. While kinetic pathways and micelle formation dynamics constitute a significant domain within soft matter and colloid science, they fall outside the scope of this article. Emphasizing this distinction is essential to avoid potential misinterpretation, as the discussed models, parameters, and experimental approaches are inherently rooted in equilibrium thermodynamics. Special attention is given to experimental methods employed in their study, including surface tension, conductometric, and calorimetric techniques, as well as to the implications of these systems in pharmaceutical applications. By integrating recent advances, this review seeks to provide a coherent framework for understanding and exploiting the unique properties of mixed micellar systems for drug delivery and formulation.

## 2. Fundamental Physicochemical Principles of Micellization

Over the past century, research on micelles has evolved from early empirical observations of the aggregation of surfactants into a complete scientific discipline that integrates relevant findings from colloid and medicinal chemistry, physical chemistry, and theoretical chemistry. Landmark developments such as the early concepts of McBain and Harkins and the advancement of modern structural analysis methods have gradually enabled the quantitative description of mixed micellization and drug–surfactant interactions. Recent advances in molecular-level structural characterization methods and computational modeling now allow for the mapping of drug localization within mixed aggregates, as well as the prediction of non-ideal mixing behavior, which in turn provides a basis for rational formulation design. The historical overview outlined below highlights how methodological advances have shifted the field from descriptive studies toward predictive, application-oriented strategies.

**Scheme 1:**
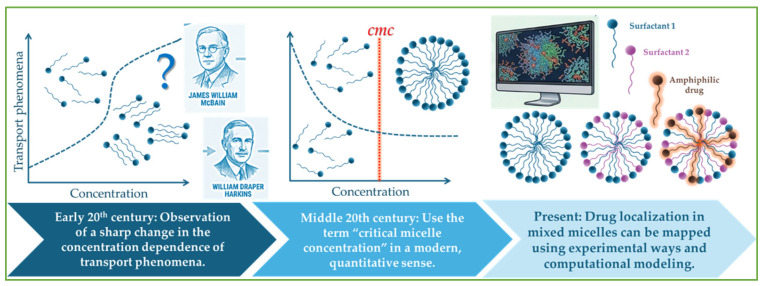
The historical evolution of research dedicated to the study of micelles from early empirical observations to modern predictive design. The schematic illustrates the century-long progression of the field, highlighting the integration of colloid, medicinal, physical, and theoretical chemistry.

Amphiphilic molecules, sometimes referred to as surfactants, spontaneously self-assemble into aggregates, or micelles in scientific terms, above a specific concentration called the *cmc*. This process is called micellization or micelle formation. The concept of the *cmc* was introduced by researchers in colloid chemistry in the early 1900s. The early foundation of the concept is credited to the British American chemist James William McBain. While the explicit term *cmc* may not have appeared in his very first articles, he was the first to describe and demonstrate that large, ionic aggregates (Scheme 1), or micelles, form in aqueous solutions of association colloids (e.g., soaps) above a specific concentration [13].

The phenomenon described by McBain was the critical concentration above which micelles form, and the term *cmc* eventually became the standard terminology in colloid chemistry. The first scientist to use the term “critical micelle concentration” in a modern, quantitative sense was William Draper Harkins [14].

Nowadays, it is well known that this occurs through the hydrophobic effect, whereby hydrophobic moieties cluster to minimize water exposure while hydrophilic headgroups remain solvated [15]. Thermodynamically, micellization is characterized by a negative Gibbs free energy (Δ*_mic_G*), indicating spontaneity, with the relative magnitudes of enthalpy (Δ*_mic_H*) and entropy (Δ*_mic_S*) determining whether aggregation is enthalpy- or entropy-driven [16]. These principles govern micellar structure, size, and stability, influencing solubilization and interfacial properties in chemical, biological, and pharmaceutical systems.

### 2.1. Driving Forces of Micellization: Hydrophobic Effect, Electrostatic Interactions, and Enthalpy–Entropy Balance

**Figure 1:**
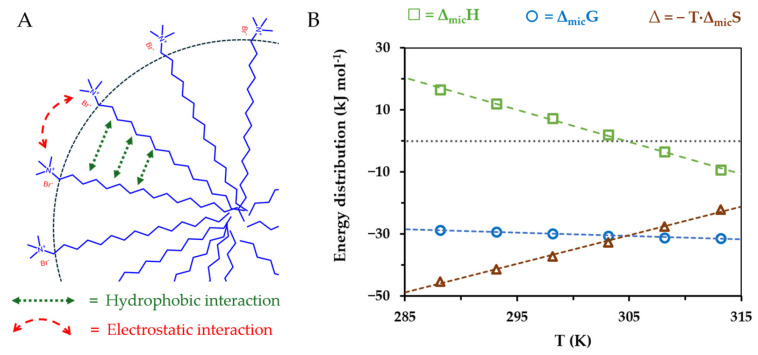
(**A**): Schematic representation of the hydrophobic interactions between nonpolar alkyl tails and electrostatic interactions between charged head groups of the cationic surfactant (cetyltrimethylammonium bromide, CTAB) molecules in a micelle. (**B**): Variation of the thermodynamic parameters for the micelle formation of cationic surfactant versus temperature [19].

The hydrophobic effect refers to the tendency of nonpolar molecules or molecular regions to aggregate in aqueous environments to minimize their contact with water, as in the schematic representation of Figure 1A [17,18]. This arises from the disruption of the hydrogen-bonding network of water and the associated entropic penalty of structuring water around nonpolar groups. When nonpolar groups cluster together, ordered water is released, increasing the system’s overall entropy. In addition, micelle formation is always a spontaneous process driven by a positive entropy gain that overcomes the enthalpy term. Enthalpy can be endothermic or exothermic, depending on the system and temperature (Figure 1B), because it involves both the unfavorable repulsion of hydrophilic heads and the favorable hydrophobic effect, making the overall balance complex. Consequently, micellization may exhibit enthalpy–entropy compensation, with the dominant driving force shifting depending on the physicochemical context.

Overall, the interplay between hydrophobic interactions, electrostatic forces, and thermodynamic contributions determines the formation, stability, and structural characteristics of micelles, providing a framework for understanding their behavior in complex surfactant systems.

### 2.2. Critical Micelle Concentration (cmc): Definition, Experimental Determination, and Interpretation

**Figure 2:**
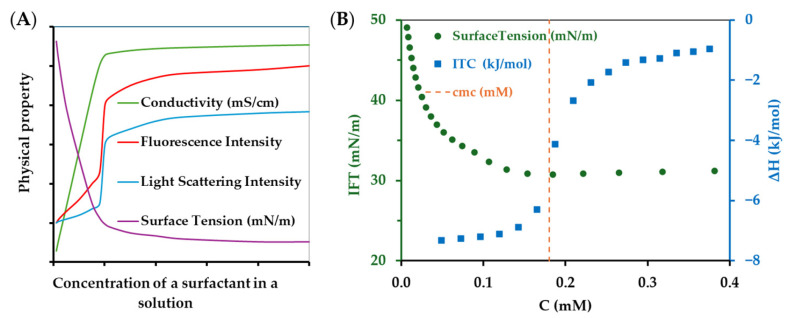
Physicochemical aspects of surfactant self-assembly and determination of the *cmc*. (**A**): Schematic representation of how measurable properties of an aqueous surfactant solution evolve with increasing surfactant concentration. Surface tension decreases rapidly at low concentrations due to interfacial adsorption and levels off after interface saturation. Bulk-sensitive properties (conductivity, fluorescence, and light scattering) increase and show a change in slope at the onset of micelle formation. (**B**): Experimental determination of the *cmc*. Surface tension (green circles) decreases and levels off with concentration, while ITC enthalpy changes (blue squares) show a distinct transition. The dashed line marks the *cmc* (~0.18 mM), where micellization begins and additional surfactant partitions into micelles rather than the interface. Redrawn from ref. [19].

The *cmc* represents a thermodynamic threshold where the free energy of micelle formation becomes lower than maintaining additional surfactants in solution [15,20,21]. Experimentally, the *cmc* can be determined using a variety of techniques that detect changes in physical properties as micelles begin to form. Common methods include tensiometry, which measures surface tension; conductivity measurements for ionic surfactants; and fluorescence spectroscopy to monitor changes in molecular environments (Figure 2A). Each method identifies a characteristic discontinuity or change in slope as the surfactant concentration crosses the *cmc* [22,23,24,25].

In addition to these methods, isothermal titration calorimetry (ITC) can be used to determine the *cmc*. ITC measures the heat released or absorbed as a surfactant solution is diluted or concentrated. When the surfactant concentration crosses the *cmc*, thermodynamic changes occur because the system shifts between monomer-dominated and micelle-dominated states. This produces a clear change in the heat of dilution, allowing the *cmc* to be extracted from the calorimetric profile (Figure 2B). ITC is an especially valuable technique because it provides not only the *cmc* but also the enthalpy of micellization, allowing deeper thermodynamic analysis of self-assembly [26,27]. Interpretation of the *cmc* provides insight into the balance of intermolecular forces driving micellization. A lower *cmc* indicates stronger hydrophobic interactions or favorable head group interactions that promote micelle formation at lower concentrations. Conversely, a higher *cmc* reflects weaker hydrophobic effects, increased head group repulsion, or environmental conditions that destabilize micelles. Factors such as temperature, ionic strength, surfactant tail length, and head group charge strongly influence the *cmc*. Overall, the *cmc* serves as a fundamental parameter connecting molecular structure, thermodynamics, and self-assembly in colloid and interface science [16,28]. Consequently, its accurate determination and interpretation are essential for optimizing surfactant-based systems in pharmaceutical, chemical, and biotechnological applications.

### 2.3. Regular Solution Theory and Rubingh Model—Non-Ideality, β-Interaction Parameter, and Activity Coefficients

The formation of mixed micelles in multicomponent surfactant systems is commonly described using regular solution theory [29] in combination with the Rubingh model [30], which together provide a quantitative framework for analyzing non-ideal mixing behavior. In an ideal mixed micelle, the surfactant composition would be calculated based on the theoretical work of Clint [31], and their interactions would be indistinguishable from those in the pure systems. Thus, in the case of a mixed micelle with ideal behavior, when surfactants are mixed, the *cmc* of the mixture (*cmc_m_*) can be calculated based on the *cmc* of each pure surfactant (*cmc*_1_ and *cmc*_2_) and the corresponding mole fractions (α) of the surfactants, as shown in Equation (1).

(1) $$\frac{1}{{cmc}_{m}}=\frac{\alpha_{1}}{{cmc}_{1}}+\frac{\alpha_{2}}{{cmc}_{2}}$$

Real surfactant mixtures deviate from ideality because differences in head group charge, tail length, or molecular geometry introduce non-ideal interaction energies. Regular solution theory captures these deviations by introducing an interaction parameter, denoted as *β*, which quantifies the extent and sign of non-ideality. A negative *β* value indicates attractive interactions between two surfactants, promoting favorable mixing and leading to a lower mixed micelle *cmc* than pure component [32].

**Figure 3:**
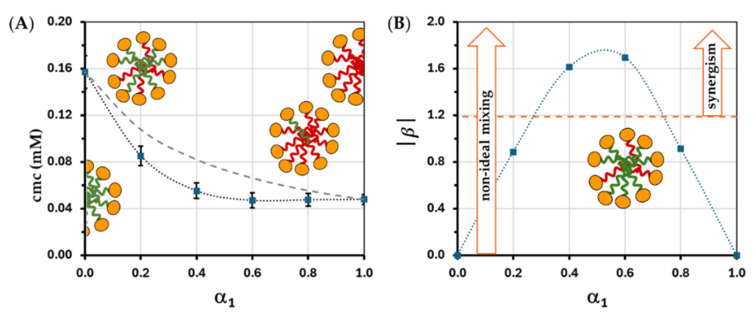
(**A**): Critical micelle concentration (*cmc*) of a binary surfactant system as a function of the mole fraction of surfactant 1. Experimental data points (blue squares) are shown with associated error bars. The dashed line represents the ideal mixing behavior predicted by Clint’s model, while the dotted line represents a fit to a non-ideal mixing model (e.g., Rubingh’s Regular Solution Theory), indicating synergistic interactions between the two surfactants. Inset drawings provide a schematic representation of the transition from pure micelles of surfactant 2 (α_1_ = 0; green) to mixed micelle structures, and finally to pure micelles of surfactant 1 (α_1_ = 1; red). (**B**): Interaction parameter (*β*) of a binary surfactant system as a function of the mole fraction of surfactant 1. Estimated *β* values (blue squares) are shown together with the secondary condition for synergism (orange dashed line). Re-edited based on ref. [33].

The Rubingh model applies regular solution concepts specifically to micellar systems, enabling the calculation of the micellar composition from experimental *cmc* values of the individual surfactants and their mixtures. Rubingh’s model considers the activity coefficients given by Equations (2) and (3)

(2) $${lnf}_{1}=\beta {\left(1-X_{1}\right)}^{2}$$

(3) $${lnf}_{2}=\beta X_{1}^{2}$$

where *f*_1_ and *f*_2_ are activity coefficients of the mixed surfactants, *X*_1_ is the mole fraction of surfactant 1 in the mixed micelle, and *β* is the interaction parameter, which considers the interaction energy between surfactants of the same (1-1) and different (1-2) structures in the mixed micelle. Figure 3 illustrates the relationship between the composition of a surfactant mixture and its ability to form micelles in a mixed micelle system.

In Figure 3, both non-ideal mixing and synergism are presented, as each describes deviations from ideal behavior that favor micellization by lowering the Clint’s model-predicted ideal value. Non-ideal mixing arises when the interactions between two surfactants differ from those predicted by ideal models (e.g., Clint’s), resulting in an experimental *cmc* lower than the ideal value. This deviation reflects attractive interactions between unlike molecules, quantified by the interaction parameter *β*. When *β* < 0 (or |*β*| > 0), the system exhibits synergism, meaning that mixed micelles are more stable than those formed by either surfactant alone. Accordingly, in Figure 3A, the experimental *cmc* values shown by the dashed curve illustrate the deviation from the predicted (calculated) values, while in Figure 3B, the synergistic range is also highlighted within the range of non-ideal micelle formation.

Importantly, non-ideal mixing does not automatically imply synergism: only those deviations from ideality that favor micellization lead to a synergistic decrease in the *cmc*. Moreover, both the magnitude of non-ideality and the appearance of synergism depend not only on the chemical structures of the surfactants but also on the composition of the solution phase, particularly the initial molar ratio of the two surfactants (α_1_). As shown in Figure 3, synergism is observed only within a specific composition window where the mixed micelles are most stabilized relative to the pure components. Thus, synergistic behavior is a phenomenon that depends to a large extent on the composition of the binary mixed surfactant system and is not merely an intrinsic property resulting from the chemical structure of the surfactant pair.

Using Rubingh’s model, one solves a set of equations relating the mixture’s *cmc* to the mole fraction of one component within the micelle, rather than in the bulk solution. Using an iterative calculation method according to Equation (4)

(4) $$\frac{X_{1}^{2}ln\left(\frac{\alpha_{1}{cmc}_{m}}{X_{1}{cmc}_{1}}\right)}{{\left(1-X_{1}\right)}^{2}ln\left(\frac{\left(1-\alpha_{1}\right){cmc}_{m}}{\left(1-X_{1}\right){cmc}_{2}}\right)}=1$$

the mixed micelle composition (*X*_1_) can be extracted from the experimental value of *cmc*_1_, *cmc*_2_ and *cmc_m_*. Since *X*_1_ is embedded in both the quadratic and logarithmic terms, the equation is transcendental and cannot be solved using basic algebra. The iteration process involves starting with an initial guess for *X*_1_ and repeatedly refining it until both sides of the equation are balanced (equal to 1), typically using numerical methods like Newton–Raphson [34] or Excel’s Goal Seek [35].

Recent studies have highlighted several conceptual and mathematical limitations of the Rubingh model [36,37,38,39,40,41,42,43], particularly regarding the internal consistency of the thermodynamic equations used to describe mixed micelle formation. As discussed in the pioneer publication of Muzzalupo et al. [44], the derivation of the fundamental relationships between micellar composition and the critical micelle concentration may omit terms that are essential for thermodynamic correctness, raising concerns about the validity of the underlying assumptions. The transcendental form of the Rubingh equation can lead to numerical and physical uncertainties, as it may yield multiple mathematical solutions and is sensitive to the input parameters. Nevertheless, the selection and determination of these input parameters do not pose practical difficulties, and reliable experimental data allow the iterative procedure to converge to physically meaningful results. Despite its limitations, the Rubingh model remains a widely used framework, provided that its assumptions and constraints are carefully considered when interpreting mixed micelle compositions.

In addition to derivation from *cmc* values, the *X*_1_ value can also be determined experimentally by small-angle neutron scattering [45], ultrafiltration [46,47], or a combination of ion-selective electrodes and ultraviolet-visible light absorption spectroscopy [48]. As a result of the iterative method and the experimental route, the fact that the micellar mole fraction (*X*_1_) may differ substantially from the bulk mole fraction (*α*_1_) demonstrates that mixed micelles can be compositionally enriched in one component. Once *X*_1_ is known, the interaction parameter *β* is calculated according to Equation (5) below,

(5) $$\beta =\frac{ln\left(\frac{\alpha_{1}{cmc}_{m}}{X_{1}{cmc}_{1}}\right)}{{\left(1-X_{1}\right)}^{2}}$$

which provides insight into intermolecular interactions that drive or hinder mixing. According to the illustrative definition of synergism, micelle formation occurs at a total mixed surfactant concentration in the solution phase that is lower than the *cmc* of both surfactants in the mixture. Corresponding to the Hua–Rosen theory [49], the minimal requirement for synergistic micellization is a negative interaction parameter

(6) β<0

which means attractive interactions between the components. However, true synergism is achieved only when the magnitude of the interaction energy exceeds the difference in the intrinsic micelle-forming tendencies of the individual surfactants, expressed as

(7) $$\left|ln\left(\frac{{cmc}_{1}}{{cmc}_{2}}\right)\right|<\left|\beta \right|$$

and indicated in Figure 3B as a dashed red line.

Another advantage of this model is that it allows the determination of activity coefficients (*f*_1_ and *f*_2_) of each surfactant within the micelle. Activity coefficients play a central role in interpreting mixed micelle stability and composition because they describe how each component’s “effective concentration” differs from its actual mole fraction [29]. For example, *f* < 1 means the surfactant is thermodynamically stabilized within the mixed micelle, while *f* > 1 indicates destabilization or exclusion. Together, *β* and the activity coefficients provide a detailed picture of the molecular forces governing mixed micellization [50]. The combined use of regular solution theory [31] and the Rubingh model has proven especially useful for mixtures of ionic and nonionic surfactants, catanionic systems, and surfactants with markedly different hydrophobic chain lengths [51]. Despite its simplifying assumptions, including the treatment of the micelle as a homogeneous phase, the Rubingh model remains a widely applied tool because it establishes a direct link between experimentally accessible quantities and microscopic interaction parameters [52].

## 3. Experimental Approaches to Study Mixed Micelles

The experimental toolkit for studying mixed micelles combines complementary methods that together reveal thermodynamic, structural, and kinetic aspects of the system. A typical strategy pairs surface and bulk measurements (*cmc* determination, surface tension, conductivity), thermodynamic probes (enthalpy, free energy), and structural techniques (size, morphology) to characterize how an amphiphilic drug integrates into surfactant micelles and how the mixture behaves. Method selection depends on the aim of the research: solubilization and partitioning require different approaches than probing internal micellar architecture. A multi-technique workflow is essential because no single method provides a complete picture; cross-validation between techniques helps separate true thermodynamic mixing from cooperative effects or kinetic limitations.

### 3.1. Conventional Surface and Bulk Methods

Conventional measuring techniques, such as tensiometry, conductometry, and spectroscopy, are rapid and relatively simple but provide limited insight into internal micellar structure and the molecular nature of interactions. The differences between the *cmc* measurement methods summarized in Table 1 arise from the fact that each technique observes different physical properties at the onset of micelle formation.

The strengths and limitations of each method, therefore, reflect the underlying physicochemical signal being monitored. Since each technique captures a different aspect of micellization, combining methods often yields the most reliable *cmc* determination.

### 3.2. Isothermal Titration Calorimetry (ITC) and Temperature-Dependent Analysis

**Figure 4:**
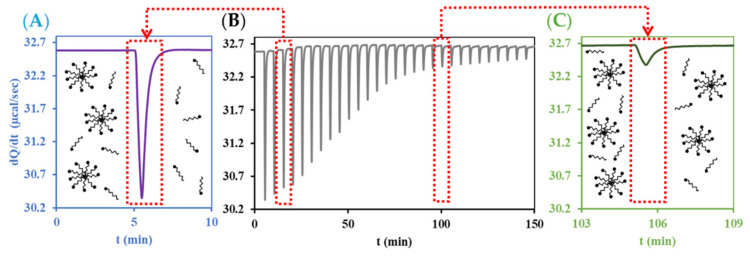
Isothermal titration calorimetry (ITC) raw data and schematic for the titration of a surfactant. (**A**) Pre-micellar regime: individual injections of surfactants into the cell produce large, sharp heat signals corresponding to the dilution and hydration of monomeric surfactant molecules, with no micelle formation. (**B**) Raw ITC thermogram showing the heat flow (μcal s^−1^) as a function of time for successive injections of surfactant. The progressive change in peak amplitude reflects the transition from solution of individual surfactant molecules dilution to micelle formation. (**C**) Post-micellar regime: once the *cmc* is exceeded, injected surfactant partitions into existing micelles, resulting in smaller, more uniform heat signals dominated by micelle dilution rather than micellization. Re-edited by ref. [19].

Isothermal titration calorimetry (ITC) directly measures heat changes during micellization and drug incorporation, delivering a detailed thermodynamic profile of the process. As shown in Figure 4, the ITC experiment begins in the pre-micellar regime, where the initial injections of surfactant micelles enter a cell containing pure solvent. At concentrations below the *cmc*, injected micelles dissociate into monomers. This process, demicellization, is accompanied by the hydration of the hydrophobic surfactant tails, which typically results in large, sharp exothermic heat signals (Figure 4A). As the titration moves through the transition region shown in Figure 4B, the amount of free surfactant monomers in the cell gradually increases and approaches the *cmc*. During this stage, the thermodynamic driving force for breaking up micelles becomes weaker because the chemical potential of the monomers in the cell starts to match that of the monomers inside the micelles. As a result, the heat pulses become smaller.

Eventually, the system reaches the post-micellar region, shown in Figure 4C. Here, the concentration in the cell is already above the *cmc*, and the solution is saturated with monomers. Therefore, newly injected micelles no longer break apart; instead, they stay intact or simply mix with the micelles already present. The heat signals become small and constant, reflecting only the enthalpy of diluting a micellar solution rather than the much larger heat effect of micelle disruption.

**Figure 5:**
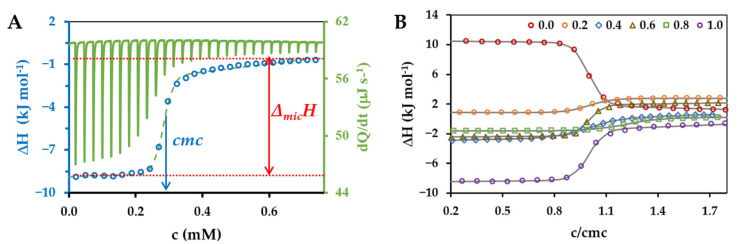
(**A**) Differential heat flow (*dQ/dt*) and enthalpy change (Δ*H*) as a function of surfactant concentration (c/mM). The blue arrow marks the *cmc*, identified by a distinct change in heat flow. The red arrow indicates the enthalpy of micellization corresponding to the space between the plateau region of the enthalpy curve. (**B**) Enthalpograms (graph of enthalpy change versus time during a reaction) of surfactant mixtures of different compositions as a function of concentration normalized to *cmc* (c/cmc). The different symbols indicate the increasing proportion of surfactant 1 (given in mole fraction); in this way, the enthalpy values marked with blue circles in parts (**A**,**B**) of the figure belong to the pure surfactant 1. Panel (**B**) is reprinted from Ref. [19] under the terms of the Creative Commons Attribution (CC BY) license.

By monitoring the heat changes as a function of surfactant concentration, the molar enthalpy of micellization (Δ*_mic_H*) can be directly determined. This is accomplished by measuring the heat released or absorbed during the stepwise titration of a concentrated surfactant solution into the solvent. As shown in Figure 5, the *cmc* and the micellization enthalpy obtained from ITC can be evaluated from the integrated heat signals.

Each injection peak is integrated to obtain heat, which is then normalized to the number of moles added. These molar enthalpy values are plotted against the total surfactant concentration (blue circles in Figure 5A), revealing two linear regions—one before micelle formation and one after. The *cmc* corresponds to the x-value at the inflection point of the sigmoidal transition between these two regions. The micellization enthalpy is calculated as the difference between the average enthalpy values before and after the *cmc*. When mixed micelles are formed, the enthalpograms change compared to those of the pure surfactants, as shown in Figure 5B. Therefore, for mixtures of different surfactants, the dependence of the *cmc* and the related thermodynamic parameters on composition can be determined from a single measurement series.

The Gibbs free energy (Δ*_mic_G*) is calculated from the *cmc* value given in mole fraction (*X_cmc_*) identified at the inflection point of the titration curve using the following relation (Equation (8)).

(8) $${∆}_{mic}G=RTln\left(X_{cmc}\right)$$

For ionic surfactants, the standard free energy of micellization can be derived directly from the *cmc* by accounting for the partial dissociation of micelles. In contrast to nonionic systems, ionic surfactants require a correction factor because a fraction of the counterions remains associated with the micellar surface. This effect is described by the degree of dissociation (*α*), which represents the fraction of counterions that remain free in solution. Incorporating this parameter, the standard free energy of micellization becomes (Equation (9)):

(9) $${∆}_{mic}G=\left(1+\alpha \right)RTln\left(X_{cmc}\right)$$

where the multiplicative factor (1 + *α*) accounts for the fact that both the surfactant ions and the dissociated counterions contribute to the overall thermodynamic balance of the aggregation process. This formulation provides a straightforward means of estimating the micellization of free energy from experimentally accessible quantities, while properly capturing the electrostatic contributions characteristic of ionic surfactant systems.

Besides the ITC measurements, the thermodynamics of micellization are derived from the temperature dependence of the *cmc*, which typically follows a U-shaped curve with a minimum near room temperature. The variation of the natural logarithm of *cmc* with temperature can be modeled using a second-order polynomial according to Equation (10) below,

(10) $$ln(cmc)=a+bT+cT^{2}$$

where *a*, *b*, and *c* are fitting constants. At constant pressure, the isosteric enthalpy of micelle formation (Δ*_mic_H_vH_*) is calculated via Equation (11) [55].

(11) $${∆}_{mic}H_{vH}={-RT^{2}\left(\frac{\partial ln(cmc)}{\partial T}\right)}_{p}$$

Finally, the entropy term (*T∆_mic_S*) and the heat capacity change (*∆_mic_C_p_*) of micellization are given by Equations (12) and (13) [56].

(12) $${T∆}_{mic}S={∆}_{mic}H-{∆}_{mic}G$$

(13) $${∆}_{mic}C_{P}={\left(\frac{\partial \left({∆}_{mic}H\right)}{\partial T}\right)}_{P}$$

The integration of temperature-dependent *cmc* data with the van’t Hoff interpretation allows for a comprehensive quantification of the driving forces behind surfactant aggregation.

**Figure 6:**
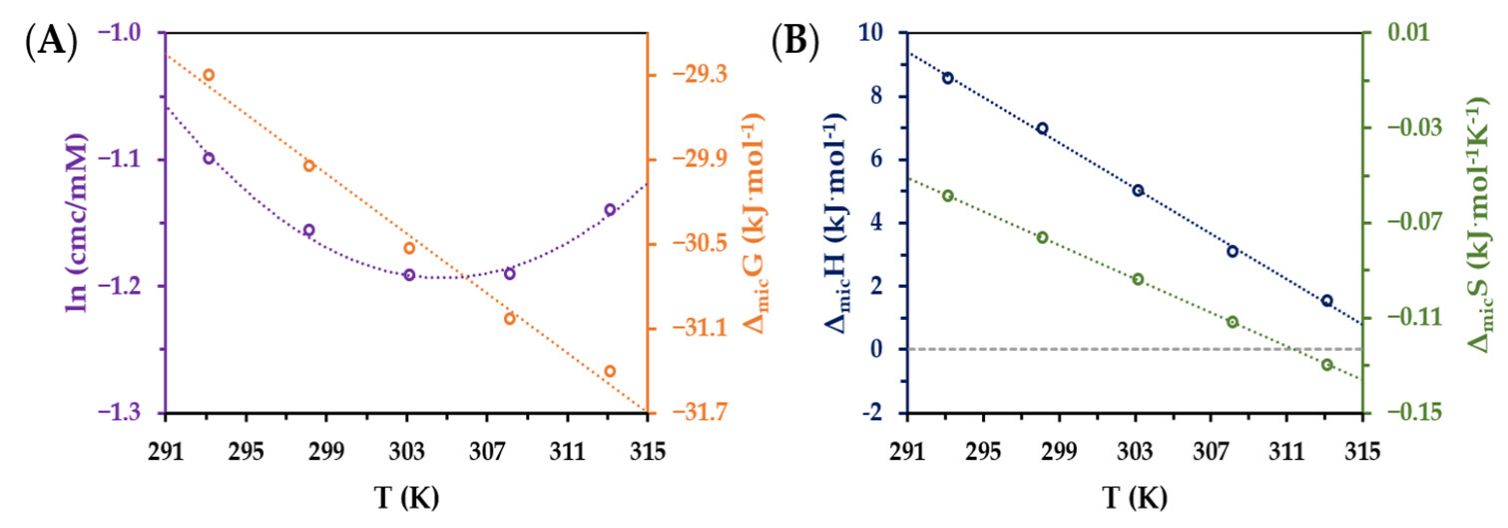
Temperature dependence of the *cmc* and the resulting thermodynamic profiles. (**A**): Experimentally determined *cmc* values and Gibbs free energy (Δ*_mic_G*) of micelle formation as a function of temperature (Δ*_mic_G* values were calculated by Equation (6)). (**B**): Temperature dependence of the enthalpy (blue circles; Δ*_mic_H_vH_*) and entropy (green circles; *∆_mic_S*) of micelle formation (heat capacity change in micellization calculated by Equation (13) as slope of the dashed blue line: *∆_mic_C_p_* = −0.34 kJmol^−1^K^−1^). Based on data of Ref. [19].

The representative experimental results in Figure 6 illustrate the characteristic temperature dependence of the *cmc*, a nonionic surfactant (octyl phenol ethoxylate), and the resulting thermodynamic profiles, highlighting the transition from entropy-driven to enthalpy-driven micellization. Naturally, Equations (10) and (11) only need to be applied if the *cmc* is not determined by calorimetry (in the case of tensiometry, conductometry, and spectroscopy-based techniques).

### 3.3. Molecular Mechanism of Mixed Micelle Formation

To perform a detailed thermodynamic analysis of mixed micelle formation, it is extremely important to obtain information about the mechanism of molecular association formation. When mapping the mechanism at the molecular level, we can use methods sensitive to changes in the chemical environment and can tell the difference between components with similar chemical properties.

**Figure 7:**
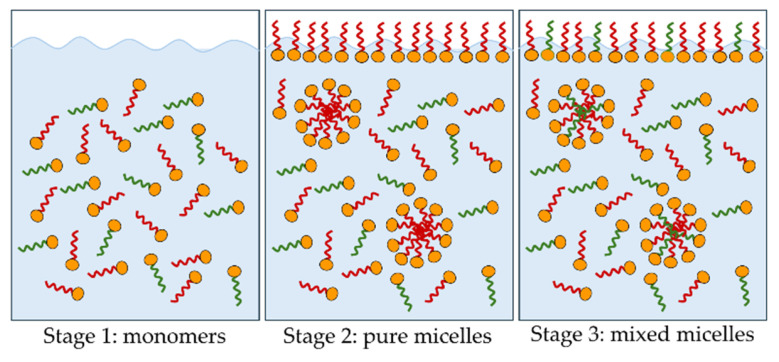
Schematic illustration of a three-stage pathway for mixed micelle assembly in aqueous solution. Stage 1: Dispersed surfactant molecules (red, nonionic surfactant) and an ionic surfactant (green) are solvated individually in water. Stage 2: Above the *cmc*, pure nonionic micelles nucleate and grow, while ionic surfactant molecules remain predominantly in solution. Stage 3: Ionic surfactant monomers subsequently incorporate into pre-formed nonionic micelles through monomer exchange and co-assembly, yielding mixed micelles containing both green and red amphiphiles.

Cui et al. provided a comprehensive molecular-level description of the formation of mixed surfactant micelles poorly understood at the mechanistic level [57]. By utilizing advanced ^1^H NMR spectroscopy and 2D NOESY experiments, the researchers demonstrated that the aggregation of surfactants in binary mixtures (including ionic/nonionic and ionic/ionic systems) does not occur synchronously. The component with the lower *cmc* in the specific mixed environment aggregates first to form pure micelles, as can be seen in the Stage 2 part of Figure 7.

Figure 7 illustrates the three-stage evolution of mixed micelles in aqueous solution. In Stage 1, both surfactants exist as independent monomers dispersed in water. No intermolecular correlations are detected in the ^1^H NMR or 2D NOESY spectra, confirming the absence of aggregation. As the total surfactant concentration increases, the system enters Stage 2, where the component with the lower *cmc* (typically the nonionic surfactant) nucleates first, forming pure micelles. This step is driven by the lower free-energy barrier for self-assembly of TX-100, whose hydrophobic chains and bulky ethoxylated headgroups promote micellization at relatively low concentration. The ionic counterpart (e.g., SDS or 12-2-12) remains monomeric, as indicated by unchanged chemical shifts and narrow resonance lines.

Upon further concentration increase, the system transitions to Stage 3, characterized by the fusion of the second surfactant into the pre-existing micelles. At this point, intermolecular cross-peaks between protons of the two surfactants appear in the 2D NOESY spectra, providing direct evidence of mixed micelle formation. The incorporation occurs through monomer exchange and cooperative interactions; electrostatic attraction between oppositely charged head groups and hydrophobic tail interdigitation stabilizes the mixed aggregates.

This sequential process demonstrates that mixed micellization is non-synchronous: the surfactant with the lower *cmc* first forms micellar nuclei, which subsequently act as templates for co-assembly of the second component. The molecular-level determinant of the Stage 1 → Stage 3 transition is, therefore, the *cmc* hierarchy coupled with favorable interfacial interactions, which govern the progression from isolated monomers to pure micelles and, finally, to energetically optimized mixed micelles containing both ionic and nonionic amphiphiles.

As the total concentration of the solution increases, the second surfactant component subsequently fuses into pre-existing micelles, resulting in the formation of mixed micellar structures. This mechanism was demonstrated across various systems, such as a mixture of quaternary ammonium dimeric surfactants and octyl-phenol ethoxylate (TX-100), where TX-100 (having the lower *cmc*) formed the initial scaffolds for subsequent fusion. The study found that close analogs, like quaternary ammonium surfactants, exhibit different behaviors based on their respective *cmc* values when mixed with TX-100. These findings indicate that mixed micelle formation proceeds via a dynamic, multi-step process rather than a single transition point [21,57].

The molecular dynamics simulations by Wei et al. align with the sequential mechanism theory by providing indirect evidence of its structural consequences [58]. The study reveals that the aggregation behavior and packing order of individual surfactants vary significantly based on their mole fractions, supporting the idea that components do not participate in the process simultaneously. Their findings indicate that the internal micellar structure and molecular arrangement are governed by the component present in a higher molar ratio. This is consistent with a model where the substance with the lower *cmc* forms the primary scaffold into which the secondary component is incorporated. The incorporation and stacking modes observed in the simulations effectively model the fusion process, illustrating how molecules of the second surfactant integrate into an established aggregate. The authors emphasize that the traditional “mixed *cmc*” concept is limited in describing these individual molecular contributions, highlighting the necessity of modern simulation techniques to accurately resolve the mechanism [58].

### 3.4. Structural Characterization of Mixed Micelles by Dynamic Light Scattering and Small-Angle Neutron Scattering Techniques

Dynamic light scattering (DLS) provides a rapid and non-invasive means of determining the hydrodynamic size and size distribution of mixed micelles in dispersed aqueous phase [59]. Because the technique is sensitive to changes in aggregate dimensions, it is particularly useful for monitoring how micelle size evolves with composition, ionic strength, or drug loading, thereby offering indirect insight into the formation of mixed aggregates and the onset of synergistic interactions [60,61]. In contrast, small-angle neutron scattering (SANS) enables a far more detailed structural analysis by probing the internal architecture of mixed micelles at the nanometer scale [62]. Through contrast variation, SANS can selectively highlight the spatial distribution of individual components, allowing the determination of core–shell organization, aggregation numbers, and the relative positioning of amphiphilic drugs within the micellar framework [63]. DLS and SANS constitute a complementary toolkit: DLS assesses overall size characteristics and colloidal stability, whereas SANS elucidates the structural motifs that dictate the physicochemical behavior of mixed micellar systems [64,65,66,67].

Despite their strengths, both methods have important limitations. DLS provides only an apparent hydrodynamic diameter, which is strongly influenced by polydispersity, multiple scattering, and the presence of even trace amounts of larger aggregates; as a result, it cannot resolve internal micellar structure or distinguish between different types of mixed assemblies. SANS, while structurally far more informative, requires access to specialized facilities and relatively high sample concentrations to achieve meaningful contrast. Furthermore, the interpretation of SANS data relies on model-dependent fitting, meaning that structural conclusions are only as reliable as the assumptions built into the chosen model. These limitations highlight the importance of combining scattering techniques with complementary physicochemical methods to obtain a robust and comprehensive picture of mixed micelle formation.

In addition to scattering techniques, zeta (ζ) potential measurements offer valuable complementary information for characterizing mixed micelles [68], particularly in systems containing ionic surfactants or charged amphiphilic drugs. The ζ-potential reflects the electrostatic potential at the slipping plane of the micelle and is highly sensitive to changes in surface composition, ion binding, and drug incorporation [69,70]. As such, it provides an indirect yet informative probe of surface interactions, colloidal stability, and the extent of charge compensation or redistribution upon mixed micelle formation. Monitoring ζ-potential as a function of composition, pH, or ionic strength can therefore help elucidate how electrostatic contributions influence micellar architecture and aggregation behavior under various physicochemical conditions. This parameter is especially useful when interpreting DLS and SANS data, as changes in surface charge often correlate with shifts in hydrodynamic size, aggregation number, or internal organization [71].

## 4. Mixed Micelles Between Amphiphilic Drugs and Classical Surfactants—Case Studies

Mixed micelles, in which one component is an amphiphilic drug, offer advantages such as improved drug solubility, modified release profiles, and altered biodistribution. However, treating the drug as a surfactant presents challenges that require careful experimental characterization.

The following studies illustrate how the previously described methods are applied to real systems: *cmc* and partitioning studies by tensiometry and spectroscopy, thermodynamic analysis by ITC, and structural and spectroscopic mapping of drug localization. These examples highlight practical considerations in designing mixed-micelle formulations and interpreting experimental data.

In addition to classical *cmc*-based analyses, understanding mixed micelles that contain amphiphilic drugs requires consideration of the molecular mechanism of mixed micellization. As demonstrated in Figure 7, mixed micelles rarely form through a single cooperative transition; instead, they follow a sequential, multi-stage pathway in which the component with the lower *cmc* nucleates first (Stage 2), and only subsequently incorporates the second component through monomer exchange and co-assembly (Stage 3). This mechanistic framework is essential for interpreting drug–surfactant systems, because the relative *cmc* values, aggregation propensities, and interaction parameters (*β*) determine which species acts as the “primary scaffold” and which one behaves as the “secondary incorporant”. Likewise, the thermodynamic parameters discussed in Section 3 (Δ*_mic_G*, Δ*_mic_H*, Δ*_mic_S*, and Δ*_mic_C_p_*) provide the energetic basis for these sequential steps, clarifying whether incorporation of the drug into pre-formed micelles is enthalpically or entropically driven. The following case studies, therefore, integrate mechanistic and thermodynamic interpretations to provide a deeper understanding of how amphiphilic drugs participate in mixed micelle formation.

### 4.1. Mixed Micelles in Tetracaine Hydrochloride (TCH) and Gemini Surfactant Containing Aqueous System

**Scheme 2:**
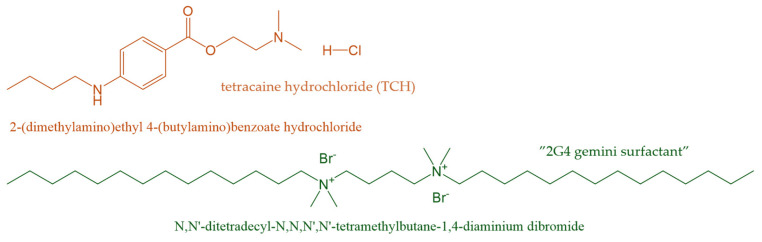
Name and chemical structures of the amphiphilic drug molecule (tetracaine hydrochloride) and the surfactant (“2G4”) used in the investigation of Azum et al. [72].

Azum et al. pointed out the case of tetracaine hydrochloride (anesthetic amphiphilic drug, TCH) mixed with cationic gemini surfactant (Scheme 2), tensiometry, and conductometry effectively track *cmc* shifts that may reflect drug incorporation in mixed micelles [72].

The aim of the presented work was to develop novel mixed micelles and characterize their thermodynamic and structural properties, which can be used in the future as drug carriers that are less toxic and more cost-effective. The appearance of gemini surfactants often exhibits stronger cooperativity and lower *cmc*, which can enhance TCH solubility and possible embedding within micelles. Based on the interpretation of the authors, the following two conclusions can be drawn directly from the measurement data.

1. The *cmc* of the pure amphiphilic drug (93.00 mM) was consistent with values reported in the literature, whereas for mixed micelles, the experimental *cmc* obtained was lower than those predicted by Equation (1), indicating non-ideal behavior.
2. Negative *β* parameters indicate a mixed micelle formation and synergism observed with the mixed system due to the hydrophobic character of gemini molecules.

There is no doubt that since 1994, thanks to Matsuki et al. [73], a value of nearly 130 mM has been known. This article also mentions a *cmc* value of around 93 mM, but this refers to dibucaine hydrochloride rather than TCH. The higher *cmc* value is confirmed by a review article on the self-assembly and interaction of surface-active drugs, which also mentions the value of 130 mM [74] based on Matsuki’s work [73]. However, in a lesser-known and less frequently cited earlier study, Shaikh and Matsuki indicate a different value for the drug, namely 99.8 mM [75].

**Figure 8:**
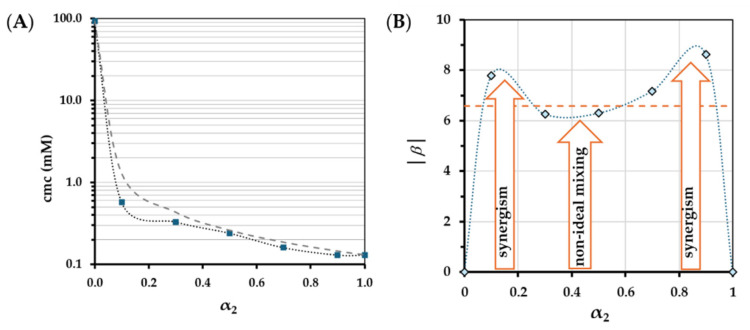
Mixed micelle formation in tetracaine hydrochloride (TCH)/gemini surfactant system. (**A**): Critical micelle concentration of pure and mixed amphiphilic molecules as a function of the mole fraction of the gemini surfactant (dashed grey line represents the ideal *cmc* values). (**B**): Absolute values of the interaction parameter (|*β*|) as a function of the mole fraction of gemini surfactant (orange dashed line represents the value of the second condition for synergism: |ln(*cmc*_1_/*cmc*_2_)|). Dotted blue lines are only guides. Based on data of Ref. [72].

The results of independent test series conducted in the meantime (i.e., between 1994 and 2022) showed significant differences in the *cmc* value of the TCH. Based on the variation of the surface tension (γ) of aqueous solutions of TCH as a function of concentration, the value of 83.4 mM was found by Zelmat et al. [76]. To investigate micellization and structural interactions in aqueous solutions of TCH, Vasim et al. performed experimental measurements at a temperature of 298.15 K and found that the *cmc* value of the drug was 170 ± 20 mM [77]. Based on the information currently available, it can only be concluded that the relevant *cmc* value can vary within extremely wide limits. This also confirms that the value proposed by Azum et al. may be correct, but the most convincing evidence for this would have been the measurement they carried out with the pure amphiphile drug, which has not been reported in their article [72]. The graphs in Figure 8 illustrate why the *cmc* value of pure surfactants (and/or amphiphiles) is a key parameter in terms of the properties of mixed systems.

Looking at the results summarized in Figure 8, it can be concluded that although negative *β* parameters are indeed observed across the entire composition range, favorable mixed micelle formation can only be observed in two separate ranges. The condition (|ln(*cmc*_1_/*cmc*_2_)| < |*β*|) represented by the dotted red line in part B of Figure 8 must also be considered. Therefore, it is not correct to conclude that synergism was observed in the gemini/TCH mixed system.

Another very important clarification is needed regarding the TCH/gemini system, which stems from the solubility of the drug. Solubility of TCH in water at 20 °C and above is larger than 100 mg/mL [78]. This value is perfectly adequate because solutions between 0.5% and 1% are used in clinical applications [79,80]. The “solubilization” of TCH with gemini surfactant does not serve its dissolution, but to optimize its therapeutic index. For example, toxicity can be reduced by decreasing the free fraction, while efficacy can be increased by micellar storage and enhanced penetration.

From the perspective of the three-stage mechanism illustrated in Figure 7, the TCH/gemini system clearly follows a sequential assembly pathway. Because the gemini surfactant possesses an extremely low *cmc* (10^−1^–10^−2^ mM range), it is the component that reaches Stage 2 first, forming pure gemini micelles long before TCH begins to aggregate. The drug therefore enters Stage 3 as the “secondary incorporant”, joining pre-existing gemini aggregates through hydrophobic and cation–π interactions. This interpretation is fully consistent with the strongly negative *β* values and the large negative deviations from ideal *cmc* predicted by the Clint model. Thermodynamically, the highly negative ΔG° values reported for the TCH–gemini mixtures indicate that incorporation of TCH into gemini micelles is spontaneous and entropically favored, reflecting dehydration of the drug’s aromatic moieties and the release of structured water. The fact that TCH has a much higher and experimentally variable *cmc* explains why it cannot form Stage 2 aggregates under the conditions studied; instead, it is recruited into gemini micelles only after the gemini scaffold has formed. Thus, the TCH/gemini system represents a textbook example of Stage 3 incorporation governed by strong hydrophobic complementarity and favorable mixing energetics.

The structural basis for this behavior lies in the fact that a gemini surfactant containing two long hydrophobic chains and a flexible linker (Scheme 2) inherently has a low *cmc*; in this way, nucleation dominates in Stage 2. TCH, which has only a single aromatic ring and a tertiary amine, provides insufficient hydrophobic surface for self-assembly and consequently enters the aggregates exclusively during Stage 3 incorporation. The strongly negative *β* values and the favorable Δ*S*° reflect the efficient dehydration of the aromatic moiety of TCH and its complementary packing within the gemini micellar core.

### 4.2. Mixed Micelle Formation from Aqueous Solution of Propranolol Hydrochloride and Cetyltrimethylammonium Bromide

**Scheme 3:**
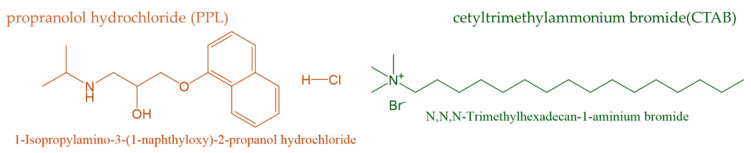
Name and chemical structures of the amphiphilic drug molecule (propranolol hydrochloride (PPL)) and the surfactant (cetyltrimethylammonium bromide (CTAB)) applied in the reported work of Bagheri et al. [81].

Bagheri et al. [52] reported in a detailed way that the propranolol hydrochloride (PPL) and cetyltrimethylammonium bromide (CTAB) system (Scheme 3) exemplifies how an amphiphilic drug modifies a classical cationic surfactant micellization. Namely, the *cmc* values shift, and mixing parameters such as the *β* parameter quantify interaction strength. In this study, the *cmc* values of pure components (CTAB and PPL) and their binary mixtures at different mole fractions were determined by plotting specific conductivity against concentration. Negative *β* values indicate synergistic interactions, while positive values suggest antagonism; these directly affect drug loading capacity and micelle stability.

**Figure 9:**
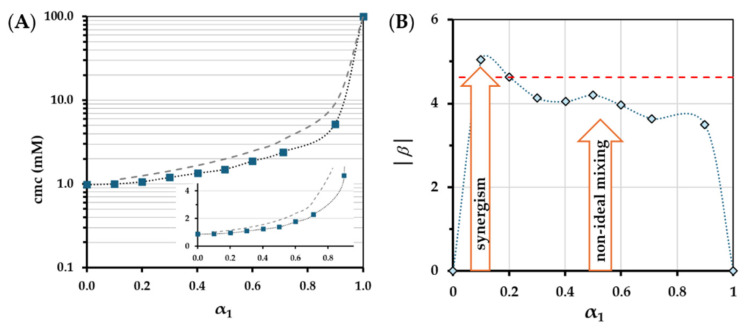
Mixed micelle formation in propranolol hydrochloride (PPL)—cetyltrimethylammonium bromide (CTAB). (**A**): The *cmc* of pure and mixed amphiphilic molecules as a function of the mole fraction of the drug (dashed grey line represents the ideal *cmc* values). (**B**): Absolute values of the interaction parameter (|*β*|) as a function of the mole fraction of PLL (red dashed line represents the value of the second condition for synergism: |ln(*cmc*_1_/*cmc*_2_)| < |*β*|)). Dotted lines are only a guide for the eyes. Based on the data of Ref. [81].

To investigate micellization in aqueous solutions of PPL, the authors performed conductometric experiments and found that the *cmc* value of the drug was 99.6 ± 0.4 mM. In earlier studies, ultrasound velocities as a function of PPL molality have shown that the *cmc* of PPL is 131 mM [82]. Although other information is available regarding the *cmc* value of the drug (108 mM [83] or 68 mM [84] based on inflections in surface tension; 130 mM based on inflections in specific conductivity [85]), the authors considered the experimental value (99.6 mM) to be correct, as it was also used by Bagheri in another study [86]. The data in Figure 9 illustrates the alternation of *cmc* and *β* values obtained from conductometric measurements as a function of the mole fraction of PLL.

Beyond the lack of synergistic effect, it is perhaps more important to consider that PPL is a freely water-soluble compound (~30 mg/mL), while its free base form has significantly lower solubility and is slightly soluble in aqueous media [87,88]. Although the physicochemical characterization of the mixed micelles consisting of PPL and CTAB presented in the article is scientifically indisputable, the solubility of the drug salt in water raises significant questions about the pharmaceutical necessity and practical usefulness of micellar solubility.

The PPL/CTAB system also aligns well with the mechanistic model of Figure 7**.** CTAB, with its *cmc* near 1 mM, reaches Stage 2 first and forms the initial micellar scaffold. PPL, whose *cmc* is one to two orders of magnitude higher, remains predominantly monomeric in solution until CTAB micelles are already present. The negative *β* values observed across the composition range, therefore, reflect Stage 3 incorporation of PPL into CTAB micelles rather than co-nucleation. Increasingly negative Δ*G*° and the rise in counterion binding (α) with CTAB mole fraction indicate that PPL incorporation stabilizes the CTAB micelle by reducing electrostatic repulsion and enhancing hydrophobic packing. Because PPL is freely water-soluble, its incorporation is not driven by solubility limitations but by favorable entropic contributions associated with dehydration of its naphthalene ring system. Thus, the PPL/CTAB system exemplifies a mechanism in which the classical surfactant dictates the onset of aggregation (Stage 2), while the amphiphilic drug participates only after micelles have formed (Stage 3).

Long alkyl chain and quaternary ammonium headgroup (Scheme 3) of cationic surfactant ensure early Stage 2 micellization, whereas PPL (despite its amphiphilic naphthalene ring system) has a much higher *cmc* and remains monomeric until Stage 3. The negative *β* values and favorable Δ*G*° arise from dehydration of the naphthalene core and from reduced electrostatic repulsion as PPL associates with the CTAB headgroup region. These structural features explain the entropically driven incorporation and the rise in counterion binding.

### 4.3. Micellization of Tricyclic Drugs with Conventional Surfactants

**Scheme 4:**
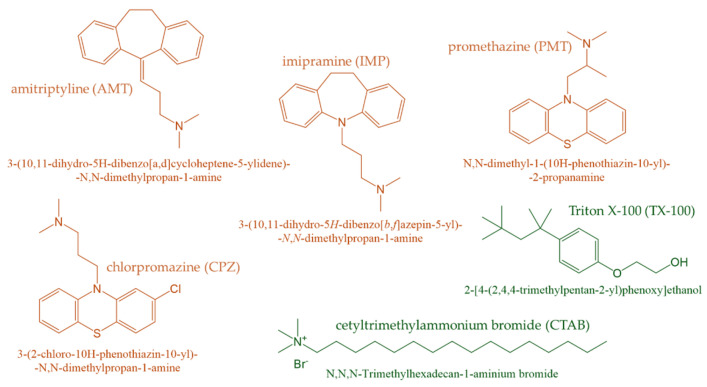
Name and chemical structures of amphiphilic drug molecules (amitriptyline: AMT, imipramine: IMP, chlorpromazine: CPZ, and promethazine: PMT) and surfactants (cetyltrimethylammonium bromide: CTAB and polyethylene glycol t-octylphenyl ether: TX-100) applied in the reported article of Alam et al. [5].

Alam et al. investigated the micelle formation and the surface properties of four amphiphilic drugs (amitriptyline: AMT, imipramine: IMP, chlorpromazine: CPZ, and promethazine: PMT) in water and in the presence of varying concentrations of NaCl, CTAB, and Triton X-100 (Scheme 4) [5].

**Figure 10:**
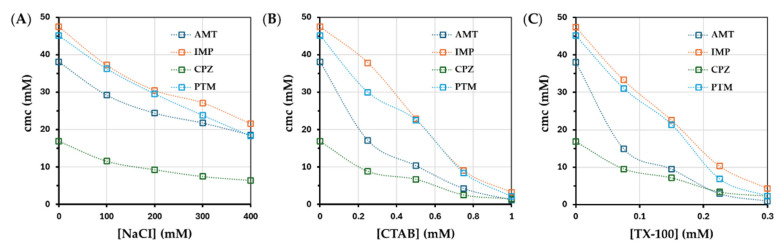
Effect of concentrations of additives ((**A**): NaCI, (**B**): CTAB, (**C**): TX-100) on the *cmc* of the amphiphilic drugs (amitriptyline: AMT, imipramine: IMP, chlorpromazine: CPZ, and promethazine: PMT). Dotted lines are only a guide for the eyes. Based on the data of Ref. [5].

Due to the unique approach of the series of experiments, the composition range examined does not cover the entire mole fraction region. Surfactant added to tricyclic drugs only reached lower concentrations; thus, only a mole fraction range between 0 and a maximum of 0.02 was examined. The *cmc* values were determined by surface tension and the dye solubilization method [89], and these systems showed the same behavior in the presence of additives. NaCl decreases critical micelle concentration, according to the authors. Counterions bind to micelles, reducing electrostatic repulsion between charged head groups. While CTAB and TX-100 have similar numerical trends for *cmc* to NaCl, the behavior is affected by mixed micelles between the drugs and surfactants (Figure 10).

**Figure 11:**
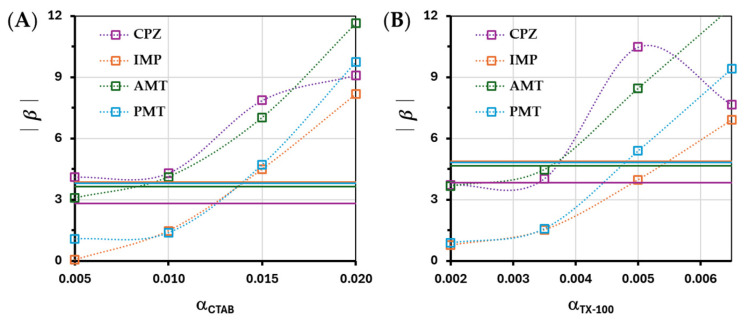
Absolute values of the interaction parameter (|*β*|) of mixed micelles against the mole fraction of additives ((**A**): CTAB, (**B**): TX-100), the amphiphilic drugs (amitriptyline: AMT, imipramine: IMP, chlorpromazine: CPZ, and promethazine: PMT), and surfactant-containing systems. Solid lines represent the value of the second condition for synergism: |ln(*cmc*_1_/*cmc*_2_)|). Dotted lines are only a guide for the eyes. Based on the data of Ref. [5].

Additionally, the investigated drug/surfactant systems show an increase in synergism as the surfactant concentration increases, as is indicated in the graphs of Figure 11.

The absolute value of the *β* parameter increases with the amount of surfactants, which demonstrates the change in the degree of interaction between the individual amphiphilic drugs and the surfactants.

The rate of increase varies between the individual active ingredients and surfactants and cannot be considered linear, reaching and exceeding the value confirming the presence of synergistic effects, which is shown by the continuous lines in Figure 11. The presented work proves that thermodynamically driven interactions lead to the formation of mixed micelles, which improve efficacy by reducing the free drug fraction.

The mixed systems formed between tricyclic drugs and CTAB or TX-100 also follow the sequential pathway shown in Figure 7. In all cases, the surfactant (CTAB or TX-100) has a substantially lower *cmc* than the drug, meaning that Stage 2 is dominated by the surfactant, which forms pure micelles before any drug aggregation occurs. The observed decrease in drug *cmc* in the presence of additives, together with the increasingly negative *β* values, reflects the progressive Stage 3 incorporation of drug molecules into these pre-formed micelles. The thermodynamic implications are consistent with this mechanism: the enhanced *Γ_ma_*_x_, reduced *A_min_*, and increased solubilization capacity indicate that incorporation of the drug into the micellar core is entropically favorable, driven by dehydration of the tricyclic aromatic rings and by reduced headgroup repulsion in the presence of CTAB or TX-100. Because the drug mole fraction never approaches the regime where drug-rich micelles could form, the system never enters a co-nucleation regime; instead, it remains firmly within the Stage 3 incorporation domain. This explains why synergism increases monotonically with surfactant concentration and why the surfactant dictates the micellar architecture throughout.

The tricyclic drugs (Scheme 4) share a rigid, polyaromatic scaffold that provides a large hydrophobic surface ideally suited for Stage 3 incorporation. CTAB and TX100, each with a low *cmc* arising from either a long alkyl chain or a bulky ethoxylated head group, dominate Stage 2 nucleation. The negative *β* values, reduced *A_min_*, and increased *Γ_max_* reflect tight packing between the tricyclic rings and the micellar core, consistent with an entropically favorable dehydration process that stabilizes the mixed aggregates.

### 4.4. Micellization of Amitriptyline Hydrochloride with Conventional and Gemini Surfactants

**Scheme 5:**
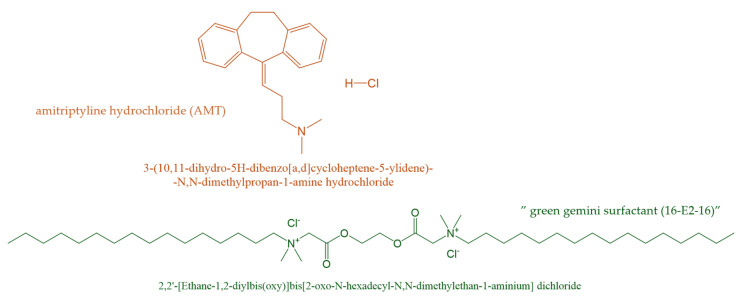
Name and chemical structures of amphiphilic drug molecule (amitriptyline hydrochloride: AMT) and green gemini surfactant (16-E2-16) applied in the reported research of Rub et al. [93].

Micelle formation of an amphiphilic drug (amitriptyline hydrochloride: AMT) with conventional and gemini surfactants has been studied by Kabir-ud-Din et al. Measurements were carried out in pure and mixed states in aqueous solutions at varied temperatures to derive several physicochemical properties. According to the authors’ statements, all the results indicate synergism and attractive interactions in the mixed systems [90]. The correlation between specific conductivity and AMT concentration at a temperature of 298.15 K showed that the *cmc* value of the active substance corresponds to 32.60 mM. At this temperature, AMT has a solubility of 10.84 mg L^−1^ in water, and it has a pKa of 9.4 [91], which means that in a solution with neutral pH, molecules are positively charged and more water-soluble [92]. As a result, improving solubility is not a priority in this situation, despite the fact that the pharmaceutical industry uses micelles to improve the absorption of many drugs in the human body. AMT is used to treat depression and, in addition to the desired effects, it also has several undesirable consequences. When drugs are used in combination with a suitable drug carrier, such as green/biocompatible gemini surfactants, these negative side effects are reduced. Rub et al. examined the interaction among the same antidepressant drug (AMT) and a green gemini surfactant (16-E2-16, Scheme 5) through surface tension measurement and fluorimetry-based techniques in different aqueous (pure aqueous/electrolyte/urea) solutions [93].

A concentration of 32.63 mM was determined, which is approximately the same as the value determined by Kabir-ud-Din et al. using conductometry [90]. The authors concluded that the *cmc* values of the mixtures were below the estimated ideal mixing estimated values, which proves the attractive interaction between the tested components (drug and gemini).

**Figure 12:**
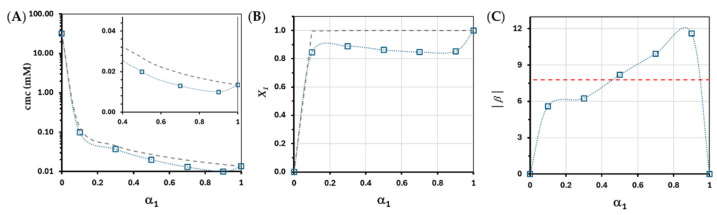
Mixed micelle formation in amitriptyline hydrochloride (AMT) and green gemini surfactant-containing aqueous systems. (**A**): Critical micelle concentration of pure and mixed amphiphilic molecules (AMT) as a function of the mole fraction of the gemini surfactant (dashed grey line represents the ideal *cmc* values). (**B**): Mole fraction of the gemini surfactant in the mixed micelle (*X*_1_) as a function of the mole fraction of the gemini surfactant in the aqueous bulk (α_1_) phase (dashed grey line represents the ideal *X*_1_ values). (**C**): Absolute values of the interaction parameter (|*β*|) as a function of the mole fraction of the gemini surfactant (red dashed line represents the value of the second condition for synergism: |ln(*cmc*_1_/*cmc*_2_)|). Dotted lines are only a guide for the eyes. Based on the data of Ref. [93].

Figure 12 illustrates the analysis of the *cmc* of both pure and mixed amphiphilic molecules as a function of the mole fraction of gemini surfactants, highlighting the deviations from ideal behavior. Furthermore, Figure 12 examines the mole fraction of the surfactant within mixed micelles and the absolute values of the interaction parameter, providing key insights into the synergistic conditions of the binary mixture. The plot of the *cmc* values as a function of the bulk mole fraction in Figure 12A clearly illustrates the non-ideal behavior of the system. This representation obscures the difference between the theoretical and experimental values, illustrated in the insert. The experimental values (blue squares) fall below the dashed line, the theoretical values calculated from the ideal Clint model (dashed grey curve). This negative deviation is particularly dramatic in the gemini-rich regions, indicating that the mixture requires a much lower concentration to form micelles than expected from a simple additive effect. The inset highlights that the minimum *cmc* is achieved at α_1_ = 0.9, where the synergy between the gemini surfactant and the drug is potent.

Part B of Figure 12 shows the change in composition of the mixed micelle as a function of the composition of the solution phase for the gemini surfactant (α _1_). According to the ideal mixing model (Clint’s theory; indicated by a dashed grey line in Figure 12B), the gemini surfactant is significantly more efficient due to its much lower *cmc*, and should dominate the micellization process. In this idealized scenario, the gemini molecules would form pure micelles themselves, leaving the surface-active drug in the bulk solution. Based on experimental data and Rosen’s theory, the reality is quite different: the drug molecules are present in the mixed micelles at a much higher proportion than predicted (e.g., 22% in reality versus the negligible 0.2% predicted by the ideal model at α = 0.1). This discrepancy can only be explained by a strong attractive interaction between the gemini surfactant and the drug molecules. The gemini surfactant effectively “pulls” the drug molecules into the micellar structure. This occurs because the two components form a thermodynamically more stable, mixed structure than they would individually, resulting in a significant synergistic effect and a highly negative interaction parameter (*β* = −5.61 at α_1_ = 0.1).

Figure 12C displays the interaction parameter (|*β*|), which quantifies the strength of attraction between the gemini surfactant and the drug. The | *β* | values peak near α_1_ = 0.9, which confirms a very strong synergistic interaction, likely due to the efficient packing of the drug molecules between the spacer groups and hydrophobic tails of the gemini surfactant. The fact that the curve stays well above the dashed reference line proves that the stabilization gained from mixing these two specific components is exceptionally high. Additionally, the lack of synergy under the reference value (|ln(*cmc*_1_/*cmc*_2_)|) does not mean that the production of a mixed system cannot have a favorable practical effect. In conclusion, the results lie not only in the fact that they demonstrate the correctness of considerations, but also that, due to the selected green surfactants, practical application is much more likely.

The AMT/gemini system provides one of the clearest experimental realizations of the mechanism depicted in Figure 7. The gemini surfactant 16-E2-16 has an exceptionally low *cmc* (10^−2^ mM), ensuring that it reaches Stage 2 almost immediately and forms the primary micellar scaffold. AMT, with a *cmc* near 30 mM, cannot nucleate micelles under these conditions and, therefore, enters the process exclusively through Stage 3 incorporation. The strongly negative *β* values (down to –11.6) and the dramatic negative deviations from ideal *cmc* confirm that AMT is thermodynamically “pulled” into the gemini micelle, forming a mixed aggregate that is far more stable than either pure component. The thermodynamic parameters (Δ*_mic_G*, Δ*_mic_H*, Δ*_mic_S*) further indicate that incorporation is entropy-dominated, consistent with dehydration of AMT’s tricyclic ring system and favorable packing between the drug and the gemini spacer region. The unusually high AMT content in the mixed micelle (far above the ideal prediction) reflects the strong attractive interactions that drive Stage 3 co-assembly. Thus, the AMT/gemini system exemplifies a highly cooperative, thermodynamically reinforced Stage 3 mechanism in which the gemini surfactant dictates micelle nucleation and the drug is incorporated only after the scaffold has formed.

The pronounced synergism in AMT/gemini systems (Scheme 5) stems from the combination of the gemini surfactant’s extremely low *cmc* and AMT’s extended tricyclic aromatic surface. The gemini micelle forms the Stage 2 scaffold, while AMT enters during Stage 3 through strong hydrophobic and spacer-mediated packing interactions. The highly negative *β* values and large positive Δ*S*° reflect extensive dehydration of AMT’s aromatic rings and the exceptional compatibility between the drug and the “gemini core”, resulting in mixed micelles far more stable than either pure component.

## 5. Advanced Technologies, Emerging Innovations, and Pharmaceutical Applications

### 5.1. Emerging Analytical and Microfluidic Innovations in Mixed Micelle Design

Looking forward, a more rigorous physicochemical understanding of amphiphilic drug–surfactant interactions opens several promising directions for the development of advanced micellar systems. Emerging analytical and computational tools may enable real-time mapping of drug distribution within mixed aggregates, supporting predictive models that surpass classical ideal-mixing assumptions [94,95,96]. Another important opportunity lies in designing mixed micelles in which the drug itself contributes to structural stability, enabling highly efficient, carrier-minimized formulations [97,98,99]. Furthermore, integrating amphiphilic drugs with biocompatible or stimuli-responsive surfactants (e.g., pH-, redox-, or enzyme-sensitive systems) may yield micellar assemblies capable of targeted or on-demand drug release [100,101,102].

Beyond molecular-level design strategies, process-intensification technologies also offer transformative opportunities. A highly promising direction is the application of microfluidics and lab-on-chip technologies for the controlled production of nanoparticles as well as mixed micelles [103]. These platforms enable rapid, diffusion-limited mixing under laminar flow, allowing precise control over micelle size, composition, and polydispersity parameters that are often difficult to regulate in conventional bulk preparation [104]. Recent studies demonstrate that microfluidic hydrodynamic focusing can generate micelles with narrower size distributions and improved reproducibility, while lab-on-chip devices facilitate real-time monitoring of micellization kinetics and drug incorporation through integrated optical or scattering-based detection modules [105,106].

Translating these physicochemical insights, advanced computational models, and novel preparation routes into in vivo relevant design principles could drastically accelerate the development of next-generation therapeutic systems. In this context, mixed micellar nanocarriers have emerged as a cornerstone strategy for overcoming the formidable biopharmaceutical hurdles associated with poorly water-soluble drugs. By fundamentally altering the physical state and physiological disposition of encapsulated active pharmaceutical ingredients, mixed micelles directly modulate critical properties such as solubility, bioavailability, cellular barrier permeation, and ultimate therapeutic action. The distinct architecture of these systems provides an elegant solution to severe hydrophobicity by trapping lipophilic drug molecules within their inner hydrophobic core, while the outer hydrophilic shell ensures excellent dispersibility in aqueous physiological environments.

### 5.2. Recent Advances in Amphiphilic Drug–Surfactant Co-Assembly Using Nonionic Systems

Several recent studies illustrate how the structural diversity of amphiphilic drugs and surfactants governs mixed micelle morphology and thermodynamic behavior. Beyond standard single-component assemblies, the primary advantage of utilizing non-ionic/polymeric surfactants and amphiphile drugs within these systems lies in their exceptional capacity to optimize thermodynamic behavior and mitigate physiological side effects by eliminating electrostatic repulsions within the micellar Stern layer. For instance, investigations into the self-assembly of the tricyclic antidepressant drug amitriptyline hydrochloride with zwitterionic phospholipids have demonstrated that simple dilution can drive structural transitions from rodlike mixed micelles to thermodynamically stable, ultrasmall unilamellar vesicles (<20 nm), a phenomenon governed by changes in the spontaneous curvature of the constituent molecules [107].

Similarly, comparative evaluations of the antipsychotic drug chlorpromazine hydrochloride in combination with zwitterionic monomeric and gemini surfactants underscore the profound impact of structural configuration [108]. While single-chain monomers form standard spherical micelles through attractive synergistic interactions, dual-tailed gemini structures prompt the formation of larger, non-spherical aggregate networks due to heightened hydrophobicity and intense steric constraints.

The work of Naved Azum and co-workers represents a promising advancement, as they investigated the association and spectroscopic behavior of the amphiphilic drug imipramine hydrochloride (IMP) and the non-ionic surfactant TX-45 in the absence and presence of an imidazolium-based ionic liquid (1-hexyl-3-methylimidazolium chloride), utilizing various theoretical frameworks such as the Clint, Rubingh, Motomura, and Maeda models [109]. Although the study focuses strictly on physicochemical interactions, it is important to acknowledge that the broader application of ionic liquids (ILs) requires careful consideration of their environmental impact. While not uniformly toxic, many ILs exhibit significant ecotoxicological risks, motivating ongoing efforts to design functional yet environmentally benign ILs.

### 5.3. Pharmaceutical Performance and In Vivo Relevance of Mixed Micellar Nanocarriers

Collectively, these analytical, computational, and microfluidic advances establish a foundation for translating physicochemical insights into clinically meaningful design principles. As demonstrated in the research of Pandya and Singh [110], combining polymeric surfactants to form mixed micelles can significantly maximize drug solubility under challenging physiological conditions, such as the acidic environment of the stomach. This enhancement directly addresses low oral bioavailability, which is often caused by poor aqueous dissolution and extensive pre-systemic metabolism. Mixed micelles shield the entrapped drug from enzymatic breakdown and harsh chemical degradation within the gastrointestinal tract, maintaining a high concentration gradient at the absorption site. The practical impact of this modulation is robustly validated by in vivo pharmacokinetic evaluations; for instance, the oral administration of an optimized mixed micelle formulation in a Wistar rat model yielded a remarkable increase in relative bioavailability compared to a standard pure drug suspension.

To exert this systemic therapeutic action, the nanocarriers must successfully navigate biological barriers, including the dense mucosal layer and the intestinal epithelium. Mixed micelles excel in this regard due to their nanometric dimensions, typically ranging from 10 to 200 nm. Nanoparticles with a diameter below 300 nm possess the unique ability to bypass the gastrointestinal system’s mucociliary clearance mechanisms, allowing the drug-loaded micelles to efficiently diffuse through mucosal networks [111].

Finally, the ultimate therapeutic efficacy is optimized through a characteristic biphasic release profile. An initial rapid release (burst effect) occurs due to the breakdown of surface-associated micelles under sink conditions, helping the plasma concentration rapidly reach therapeutic levels. This is followed by a controlled, sustained release stage (governed by the Higuchi release model [112] and Fickian transport mechanism [113]) extending drug release up to 12 h. By maintaining steady, prolonged therapeutic levels, mixed micelles minimize peak-to-trough fluctuations, protect the drug from reticuloendothelial system uptake, maximize target site availability, and reduce non-targeted toxicities, thereby eliminating the necessity for high-dose co-medications [114].

Together, these features position mixed micelles as highly versatile and clinically promising nanocarriers for the delivery of challenging therapeutic agents.

## 6. Conclusions

The study of mixed micelle formation involving amphiphilic drugs and surfactants frequently reveals critical methodological oversights and traps that can obscure the physicochemical and pharmaceutical relevance of the findings. One common issue is the omission or inaccurate determination of pure component *cmc* data, particularly for the drug itself. Without a reliable knowledge of the micellization behavior of pure amphiphilic quantifying synergy using the Hua and Rosen-derived evaluation method, as well as Clint’s ideal models, it becomes inherently flawed.

The synergy in mixed micellar systems is frequently misunderstood. Synergism essentially means that “the whole is greater than the sum of its parts”. The micelle formation of a surfactant mixture is called synergistic if the *cmc* of the mixture is lower than we would expect based on the individual properties of the components, according to the rules of ideal mixing. Even in cases where this condition is satisfied, the limited composition ranges that have been studied are unable to capture the activity of the system and overlook the concentration points where synergistic effects are powerful.

Another unfortunate and inappropriate interpretation concerns the pharmaceutical necessity of micellar solubilization. In several studies, the drugs investigated are freely water-soluble in their salt forms without applying any formulation strategy. Applying complex micellar delivery systems to such compounds raises significant questions about practical utility, as the solubility enhancement may be redundant. In the case of poorly water-soluble active ingredients, a separate assessment must be made of studies that use mixed surfactant micelles to solubilize the active ingredient and thereby increase its solubility in water [19,115,116,117,118].

In these mixed micelles, the encapsulated molecule will accumulate in the hydrophilic core of the micelles. A completely different approach is required for the studies elaborated upon in this manuscript, which focus on mixed micelles wherein the drug integrated into the micelle functions as a micelle component.

Finally, translating these physicochemical insights and preparation routes into in vivo-relevant design principles could accelerate the development of next-generation therapeutic mixed micelles with improved stability, safety, and clinical performance.

## Figures and Tables

**Table 1:** Summary of major *cmc*-determination methods and their practical characteristics.

Method ^1^	Sensitivity to Low *cmc*	Primary Info Provided	Strengths	Limitations
Tensiometry [22,53]	Medium	Surface activity; *cmc*	Direct surface-property readout; simple	Interfered by impurities; requires clean surface
Conductometry [22]	Low	Ionization; *cmc* for ionic surfactants	Good for ionic systems; simple	Poor for nonionic or low-ionicity drugs
Fluorimetry [22,23]	High	Microenvironmental changes; *cmc*	Very sensitive; low detection limits	Probe–drug interactions can bias result
UV–vis spectroscopy [54]	Medium	Drug partitioning; apparent *cmc*	Direct drug signal if chromophore is present	Requires chromophore or dye; inner-filter effects
ITC [27]	Medium	Enthalpy, binding constants; *cmc*	Direct thermodynamic data	Concentration range of the measurement is narrow

^1^ References discuss the advantages and limitations of the methods described in detail.

## Data Availability

No new data were created or analyzed in this study. Data sharing is not applicable to this review article.
